# Comparative Performance of Artificial Intelligence and Radiologists in Detecting Lung Nodules and Breast Lesions on CT and MRI: A Systematic Review

**DOI:** 10.7759/cureus.95943

**Published:** 2025-11-02

**Authors:** Sibthein A Khalid, Tayyib Khaliq, Yousaf N Rehman, Zaynah Anwar, Waleed Mirani

**Affiliations:** 1 Orthopaedics and Trauma, Medical University of Plovdiv, Plovdiv, BGR; 2 Psychiatry, Birmingham and Solihull Mental Health NHS Foundation Trust, Birmingham, GBR; 3 Orthopaedics and Trauma, Calderdale and Huddersfield NHS Foundation Trust, Huddersfield, GBR; 4 Emergency Medicine, Frimley Park Hospital, Frimley, GBR; 5 Internal Medicine, Nishtar Medical University, Multan, PAK

**Keywords:** : artificial intelligence, breast lesions, cancer detection, ct, diagnostic accuracy, liver tumors, mammography, mri, pulmonary nodules, radiologists

## Abstract

This systematic review evaluates the comparative effectiveness of artificial intelligence (AI) and radiologists in the detection of breast lesions and pulmonary nodules using mammography, computed tomography, and magnetic resonance imaging. A comprehensive literature search identified six high-quality studies, including randomized controlled trials, prospective clinical investigations, and large-scale real-world implementation studies. Although liver imaging was included in the initial search strategy, no eligible comparative studies meeting the inclusion criteria were identified; therefore, the final analysis focused exclusively on breast and lung imaging. Across these settings, AI consistently demonstrated non-inferior or superior diagnostic accuracy compared to radiologists, with additional benefits such as reduced workload, shorter assessment times, improved triage efficiency, and enhanced predictive values. While large population-based studies confirmed the scalability of AI in cancer screening, smaller diagnostic studies highlighted its ability to support radiologists in complex cases. However, heterogeneity in study design, imaging modality, and outcome reporting, as well as concerns regarding generalizability and potential bias, underscore the need for further validation. These findings support the controlled integration of AI into clinical workflows to improve diagnostic performance and healthcare efficiency.

## Introduction and background

Medical imaging plays a pivotal role in the early detection, diagnosis, and management of cancer. Among the most commonly encountered malignancies, lung and breast cancers remain leading causes of cancer-related morbidity and mortality worldwide [1]. Accurate and timely identification of suspicious or potentially malignant lesions on computed tomography (CT) and magnetic resonance imaging (MRI) is essential for guiding further diagnostic evaluation and improving patient outcomes [2]. Traditionally, radiologists have been the cornerstone of lesion detection, relying on standardized, rigorous training and clinical experience to interpret complex imaging findings. However, the ever-growing demand for imaging studies, high workloads, and the inherent variability in case complexity and interpretive judgment among readers continue to pose challenges to maintaining optimal diagnostic accuracy and efficiency [3].

Recent years have witnessed remarkable advances in artificial intelligence (AI), particularly deep learning algorithms, in the field of medical imaging. AI systems are now capable of detecting subtle imaging features that may be overlooked by humans, thereby offering the potential to reduce inter-observer variability, enhance sensitivity for early disease, and improve workflow efficiency [4]. Numerous studies have demonstrated AI’s ability to perform at a level comparable to, and in some cases exceeding, that of experienced radiologists across a variety of imaging modalities and anatomical sites [5,6].

Despite this promise, the clinical adoption of AI in oncologic imaging requires rigorous validation. Randomized controlled trials (RCTs) and large-scale implementation studies have started to provide real-world evidence on the comparative performance of AI versus radiologists. Notably, trials in breast cancer screening have assessed whether AI can replace or augment radiologists in double-reading protocols, while investigations in thoracic imaging have examined the role of AI in detecting pulmonary nodules on CT and chest radiographs [7,8]. Similarly, AI-assisted CT techniques are being explored for optimizing nodule detection and improving diagnostic confidence in complex clinical scenarios.

However, the literature remains fragmented, with studies differing in design, patient populations, imaging modalities, and clinical endpoints. This heterogeneity underscores the need for a systematic synthesis of current evidence to clarify the diagnostic accuracy, clinical utility, and potential integration of AI into routine radiological practice for cancer detection. The aim of this systematic review is to evaluate and compare the performance of AI algorithms against radiologists in the detection of lung nodules and breast lesions on CT and MRI. Although liver imaging was included in the initial search scope, no eligible comparative studies met the inclusion criteria, and these were therefore excluded from the final synthesis. By synthesizing evidence from recent randomized trials, implementation studies, and non-inferiority analyses, we seek to determine the strengths, limitations, and clinical implications of AI-supported imaging in oncologic diagnosis.

## Review

Materials and methods

Study Design and Protocol

This systematic review was conducted in accordance with the Preferred Reporting Items for Systematic Reviews and Meta-Analyses (PRISMA) 2020 guidelines [9]. The protocol was designed prospectively, outlining the eligibility criteria, search strategy, data extraction process, and risk of bias assessment. The research question was structured using the PICO (Population, Intervention, Comparison, Outcomes) framework [10]: the population included patients undergoing screening or diagnostic oncologic imaging (mammography, CT, or MRI) for breast or lung lesion detection, the intervention was AI-assisted image interpretation, and the comparator was standard radiologist reading (single or double, depending on the study design). Diagnostic accuracy in the included studies was evaluated against either consensus readings by expert radiologists or established reference standards such as biopsy-proven or follow-up-confirmed findings. The outcomes of interest were diagnostic accuracy, sensitivity, specificity, cancer detection rates, recall rates, false positives, positive predictive value, workload reduction, and clinical or operational impact. Although liver imaging was included in the initial search to capture all potential oncologic applications, no comparative clinical studies met the inclusion criteria, and this domain was excluded from the final analysis.

Eligibility Criteria

We included RCTs, prospective clinical studies, and large-scale observational implementation studies that directly compared AI-based image interpretation with radiologists in the detection of breast lesions or pulmonary nodules. Studies were eligible if they reported primary diagnostic outcomes or system-level metrics, including workload, recall, or time-to-diagnosis. Exclusion criteria included retrospective studies without a radiologist comparator, purely technical validation studies lacking clinical outcomes, conference abstracts without peer-reviewed publication, and studies limited to non-oncologic imaging applications. Only full-text, peer-reviewed articles in English were considered. Liver imaging was part of the initial search scope to ensure a comprehensive evaluation across major oncologic sites; however, no comparative clinical studies met the inclusion criteria, as most liver-related AI papers were retrospective or lacked a radiologist comparator, and these were therefore excluded from the final analysis.

Literature Search Strategy

A comprehensive electronic search was carried out across PubMed, Embase, Scopus, and Web of Science from inception to September 2025. The strategy used both controlled vocabulary terms (Medical Subject Headings (MeSH) in PubMed, Emtree in Embase) and free-text keywords to maximize sensitivity. Search terms included “artificial intelligence”, “machine learning”, “deep learning”, “convolutional neural networks”, and “radiomics”, combined with imaging terms such as “mammography”, “computed tomography”, “magnetic resonance imaging”, and “radiology”, along with disease-specific terms including “breast cancer”, “mammary carcinoma”, “lung nodules”, and “pulmonary nodules”. Boolean operators were applied to refine the results, for example: (“artificial intelligence” OR “machine learning” OR “radiomics”) AND (“mammography” OR “computed tomography” OR “magnetic resonance imaging”) AND (“breast cancer” OR “lung nodules”) AND (“diagnostic accuracy” OR “screening” OR “cancer detection rate”). No restrictions were placed on study design or publication year. Although “liver cancer” and “hepatocellular carcinoma” were included in the initial search terms to explore hepatic imaging, no eligible comparative studies met the inclusion criteria; these were therefore excluded from the final synthesis. Reference lists of included articles and relevant reviews were also manually screened to identify additional eligible studies.

Study Selection Process

All identified records were imported into reference management software, and duplicates were removed. Two independent reviewers screened titles and abstracts for relevance. Full-text articles were subsequently assessed against the predefined eligibility criteria. Disagreements were resolved by discussion or consultation with a third reviewer. The study selection process was documented in a PRISMA flow diagram, recording the number of records identified, screened, excluded, and included in the final synthesis.

Data Extraction

Data extraction was performed independently by two reviewers using a standardized form. Extracted information included first author, year of publication, study design, sample size, population characteristics, imaging modality, comparator setup, AI system details (when available), primary and secondary outcomes, and key findings. Efforts were made to maintain consistency in the extraction process to allow cross-study comparisons.

Risk of Bias and Quality Assessment

Risk of bias was evaluated in line with the study design of each included article. RCTs were assessed using the Cochrane Risk of Bias 2.0 (RoB 2) tool [11], which considers domains such as the randomization process, deviations from intended interventions, completeness of outcome data, measurement of outcomes, and selective reporting. Diagnostic accuracy and observational studies were evaluated using the Quality Assessment of Diagnostic Accuracy Studies 2 (QUADAS-2) framework [12], which assesses bias related to patient selection, index test, reference standard, and flow and timing. For hybrid diagnostic trials with randomization components, both RoB 2 and QUADAS-2 criteria were applied as appropriate. Each study was independently rated as low risk, some concerns, or high risk of bias, and justifications were documented in accordance with PRISMA guidelines.

Data Synthesis

Given the heterogeneity in imaging modalities, cancer types, and study designs, a narrative synthesis was performed rather than a meta-analysis. Results were summarized across outcomes of diagnostic performance (sensitivity, specificity, accuracy, positive predictive value (PPV), cancer detection rates), operational metrics (reading workload, time to diagnosis, triage efficiency), and broader system-level implications. Where available, effect sizes and confidence intervals were reported to allow assessment of robustness and precision of the findings.

Results

Study Selection Process

**Figure 1:**
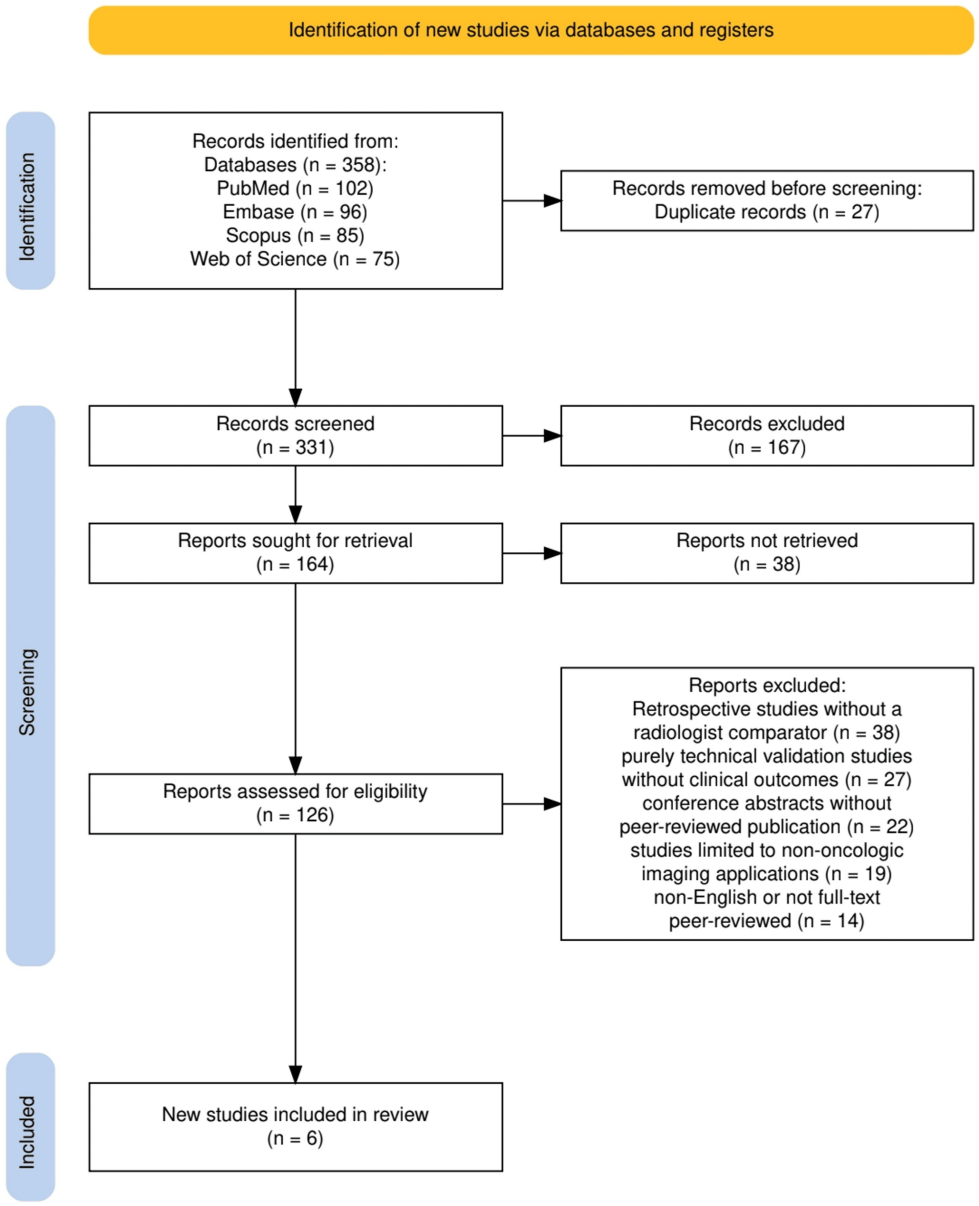
The PRISMA flowchart represents the process of the selection of the studies. PRISMA: Preferred Reporting Items for Systematic Reviews and Meta-Analyses

The study selection process is shown in the PRISMA flow diagram (Figure 1). A total of 358 records were identified through database searches. After the removal of 27 duplicates, 331 unique records were screened, of which 167 were excluded based on title and abstract. Full texts of 164 reports were sought for retrieval, and 38 could not be retrieved. Among the 126 reports assessed for eligibility, 120 were excluded for reasons including retrospective design without a radiologist comparator (n = 38), purely technical validation without clinical outcomes (n = 27), conference abstracts without peer-reviewed publication (n = 22), restriction to non-oncologic imaging (n = 19), and non-English or not full-text peer-reviewed articles (n = 14). Ultimately, six studies met the inclusion criteria and were synthesized for this systematic review.

Characteristics of the Selected Studies

The characteristics of the included studies are summarized in Table 1, reflecting a diverse set of designs, populations, and imaging modalities. Across large RCTs, prospective clinical studies, and real-world implementation research, AI was consistently evaluated against radiologists in breast and lung cancer detection. Sample sizes varied considerably, ranging from fewer than 150 patients in diagnostic lung studies to over 460,000 women in nationwide mammography screening. Modalities included mammography in breast cancer screening and CT imaging in lung cancer detection, with comparators spanning AI alone, AI combined with one radiologist, and traditional double reading. Primary outcomes focused on diagnostic accuracy metrics such as cancer detection rate, recall rate, sensitivity, specificity, and predictive values, as well as operational endpoints like workload and time to diagnosis. Overall, AI demonstrated non-inferior or superior performance compared to radiologists, with added benefits in efficiency, workload reduction, and accelerated diagnostic pathways.

**Table 1:** Characteristics of the studies included in the systematic review comparing AI and radiologists in cancer detection. RCT: randomized controlled trial; AI: artificial intelligence; PPV: positive predictive value; TA: time to additional imaging; TB: time to biopsy; CT: computed tomography; CNN: convolutional neural network; mSv.cm: Millisievert-centimeter (unit of radiation dose-length product)

Study (Author(s), Year)	Population (Sample size, demographics)	Cancer Type & Imaging Modality	Study Design	Comparator (AI vs Radiologist setup)	Primary Outcome(s)	Key Findings
Lång et al., 2023 [13]	80,033 women aged 40–80 in mammography screening	Breast cancer, Mammography	RCT, non-inferiority, single-blinded	AI-supported single/double reading vs. standard double reading	Cancer detection rate, recall rate, false positive rate, PPV, workload	AI-supported screening was non-inferior in cancer detection (6.1 vs 5.1/1000) with 44% reduced workload
Dembrower et al., 2023 [14]	55,581 women aged 40–74 in screening program	Breast cancer, Mammography	Prospective, paired-reader, non-inferiority	One radiologist + AI vs. two radiologists; AI alone; two radiologists + AI	Screen-detected breast cancer within 3 months	One radiologist + AI was non-inferior (261 vs 250 cancers). AI alone and triple reading are also non-inferior.
Eisemann et al., 2025 [15]	463,094 women aged 50–69 at 12 sites in Germany	Breast cancer, Mammography	Observational, real-world, non-inferiority	AI-supported double reading vs. standard double reading	Cancer detection rate, recall rate, PPV	AI-supported group had higher cancer detection (6.7 vs 5.7/1000; +17.6%), non-inferior recall, improved PPV recall (17.9% vs 14.9%), and biopsy (64.5% vs 59.2%).
Friedewald et al., 2025 [16]	1000 women in U.S. screening (463 AI, 392 control)	Breast cancer, Mammography	RCT, unblinded implementation study	AI triage prioritization vs. standard delayed reporting	Time to additional imaging (TA) and time to biopsy (TB)	AI triage reduced TA by 25% (6.4 days) and TB by 30% (16.8 days); all cancers were correctly prioritized by AI.
Abadia et al., 2022 [17]	103 patients with complex lung disease + 40 controls	Lung nodules, CT	Retrospective, non-inferiority study	CNN AI vs. an experienced thoracic radiologist	Sensitivity, specificity, classification accuracy, assessment time	AI sensitivity similar to radiologist (67.7%); detected 8.4% nodules missed by a radiologist; patient-level classification sensitivity 96.1%, specificity 82.5%. AI reduced assessment time (2:44 → 0:36) and increased radiologist confidence.
Zhu et al., 2023 [18]	682 patients undergoing CT (341 single-energy, 341 dual-energy)	Lung nodules, CT	Prospective RCT	AI applied to single-energy vs dual-energy CT	Sensitivity, accuracy, false-positive & miss rate, radiation dose, image quality	Dual-energy CT + AI improved sensitivity for nodule detection and image quality, with lower radiation dose (0.32 vs 0.62 mSv.cm), but accuracy was slightly poorer than single-energy AI.

Quality Assessment

The quality assessment of the included studies, summarized in Table 2, highlights generally robust methodological standards with some variability depending on study design. RCTs demonstrated a low risk of bias, supported by strong randomization processes, large sample sizes, and clearly defined endpoints, although a lack of blinding was a common limitation. Prospective non-inferiority trials also showed low risk, with minor concerns regarding awareness of AI use by radiologists. Large-scale observational implementation research was more prone to bias, primarily due to non-randomized designs and voluntary adoption of AI systems, though their extensive sample sizes provided strength in external validity. Smaller retrospective studies carried a higher risk of bias, particularly due to limited populations, potential reference standard issues, and single-expert comparators. Hybrid diagnostic trials combining randomization with diagnostic accuracy frameworks presented some concerns, especially related to reporting and lack of blinding. Overall, the evidence base is strong but not without limitations, emphasizing the need for continued rigor in trial design and execution.

**Table 2:** Quality assessment of the included studies using validated risk of bias tools. RoB 2: Cochrane Risk of Bias 2.0 tool; QUADAS-2: Quality Assessment of Diagnostic Accuracy Studies, version 2; RCT: randomized controlled trial; AI: artificial intelligence

Study (Author(s), Year)	Study Design	Tool Used	Risk of Bias Assessment
Lång et al., 2023 [13]	Randomized Controlled Trial	RoB 2	Low risk: Randomization robust, large sample, blinding of radiologists to allocation. Some concern for industry funding, but overall low bias.
Dembrower et al., 2023 [14]	Prospective, paired-reader non-inferiority trial	RoB 2	Low risk: Prospective design, adequate comparator groups. Some concern for lack of blinding since radiologists knew AI was in use, but bias is unlikely to affect outcomes.
Eisemann et al., 2025 [15]	Large multicenter, observational, real-world implementation	QUADAS-2	Some concerns: Voluntary AI adoption by radiologists may introduce selection bias. Large sample strengthens validity, but non-randomization is a limitation.
Friedewald et al., 2025 [16]	Randomized Implementation Trial	RoB 2	Low risk: Randomization, clear endpoints (time to imaging/biopsy). Open-label design (not blinded), but outcomes are objective → low risk.
Abadia et al., 2022 [17]	Retrospective diagnostic accuracy study	QUADAS-2	High risk: Small sample size (103 + 40), retrospective design, single expert comparator. Potential bias in the reference standard.
Zhu et al., 2023 [18]	Prospective RCT, diagnostic performance	RoB 2 + QUADAS-2 (hybrid diagnostic trial)	Some concerns: Randomized, but radiologists were not blinded; sensitivity improved, but accuracy was lower in the dual-energy arm. Some reporting bias is possible.

Discussion

Synthesis of Findings

Across the six included studies, AI consistently demonstrated non-inferior or superior diagnostic performance compared to radiologists across mammography and thoracic CT imaging. In breast cancer screening, large-scale randomized and real-world studies confirmed that AI either matched or exceeded radiologists in detection rates: the Mammography Screening with Artificial Intelligence (MASAI) trial reported a cancer detection rate of 6.1 vs. 5.1 per 1000 with a 44% workload reduction [13], while the PRAIM (PRospective multicenter observational study of an integrated AI system with live Monitoring) study observed a 17.6% higher detection rate (6.7 vs. 5.7 per 1000) with improved PPV of recall (17.9% vs. 14.9%) [15]. Similarly, the ScreenTrustCAD trial showed that replacing one radiologist with AI was non-inferior to traditional double reading (261 vs. 250 cancers detected) [14]. Beyond accuracy, AI demonstrated tangible workflow benefits: in Friedewald et al.'s trial, AI triage reduced time to biopsy by 30%, ensuring all cancers were prioritized [16]. Evidence from lung imaging paralleled these results; Abadia et al. found AI matched radiologist sensitivity (67.7%), detected 8.4% additional nodules, and cut reporting times from 2:44 to 0:36 minutes [17]. Zhu et al. further highlighted that dual-energy CT combined with AI improved sensitivity and reduced radiation dose [18]. Collectively, these findings underscore AI’s dual role in enhancing diagnostic accuracy and optimizing efficiency across imaging modalities.

Strengths and Limitations of the Evidence

The evidence base spans robust multicenter RCTs, real-world observational data, and focused diagnostic accuracy studies, offering breadth but also considerable heterogeneity. Strengths include the large-scale populations studied in mammography, such as PRAIM’s 463,094 participants across 12 German sites [15], which provide real-world generalizability, and MASAI’s 80,033 women, representing the first RCT of AI in screening [13]. By contrast, evidence in lung imaging remains preliminary: Abadia et al. [17] included only 103 patients, limiting statistical power, while Zhu et al. [18] examined 682 patients but within a single modality-focused trial. Methodological variability is also notable; some studies were single-blinded [13] while others were unblinded [16], potentially introducing bias. Furthermore, AI systems varied across trials (e.g., Transpara v1.7 vs. Lunit vs. proprietary CNNs), making direct comparison challenging. Importantly, most trials assessed short-term screening outcomes (cancer detection, recall, PPV), whereas long-term endpoints such as interval cancers or mortality reduction remain largely unreported. Thus, while current evidence establishes AI as diagnostically safe and potentially advantageous, limitations in study scope, heterogeneity, and follow-up constrain definitive conclusions about its sustained clinical impact.

Position Within the Broader Literature

Our synthesis complements and extends prior evidence on AI in diagnostic imaging. Earlier retrospective meta-analyses consistently highlighted AI’s potential in mammography and lung nodule detection, often reporting comparable or slightly superior sensitivity to radiologists, but these were limited by simulated workflows rather than clinical practice [19]. For example, McKinney et al. demonstrated AI’s promise in retrospective breast screening datasets, yet real-world implementation was lacking [20]. The current body of prospective and large-scale evidence, such as MASAI [13], ScreenTrustCAD [14], and PRAIM [15], provides a major step forward by demonstrating non-inferior or superior performance in actual screening programs. Importantly, these studies not only confirm earlier retrospective findings but expand the evidence base to show feasibility and safety in national-level implementation, shifting the conversation from “proof of concept” to “real-world adoption.”

Clinical and System-Level Implications

The integration of AI into imaging workflows carries profound implications for healthcare delivery. From a workforce perspective, AI may partially offset the global shortage of radiologists, particularly in low- and middle-income countries (LMICs), where the scarcity of specialized readers constrains screening programs [21]. The MASAI trial demonstrated a 44% reduction in radiologist reading workload [13], while the Friedewald study showed that AI triage shortened time to biopsy by 30%, reducing patient anxiety and accelerating treatment initiation [16]. Systemically, this operational efficiency could expand screening capacity without proportionate increases in human resources. Moreover, AI has the potential to enhance equity in access: if deployed in under-resourced settings, AI could support consistent diagnostic quality where radiologist expertise is limited [22]. However, there is a parallel risk-if AI models are trained predominantly on high-income population data, their application may inadvertently widen disparities by underperforming in diverse or underserved populations.

Risks and Challenges

Despite its promise, AI adoption is not without critical risks. Over-reliance on AI outputs could lead to diagnostic blind spots, especially if radiologists defer judgment to algorithmic results without adequate oversight. Regulatory and medico-legal questions also remain unresolved-when errors occur, it is unclear whether accountability lies with the software developers, clinicians, or healthcare institutions [23]. A further concern is generalizability: most included trials were conducted in Sweden, Germany, and the United States, with minimal data from LMIC contexts where disease prevalence, imaging quality, and clinical workflows differ substantially. Dataset bias poses another challenge; AI systems trained on homogenous populations may underperform in ethnically or demographically distinct groups, potentially exacerbating inequities. Therefore, while AI demonstrates safety and efficiency in controlled settings, its widespread deployment must be tempered with robust validation, regulatory safeguards, and post-implementation monitoring to ensure clinical safety across diverse healthcare systems.

Research Gaps

While the included studies strengthen the evidence for AI-assisted imaging, several critical gaps remain. First, there is a lack of head-to-head comparative trials across multiple modalities; most high-quality evidence focuses on breast cancer screening, whereas lung and liver applications are supported by smaller, single-center studies [24]. Notably, although liver imaging was included within the review scope, no eligible comparative studies evaluating AI performance against radiologists in hepatic lesion detection met the inclusion criteria, reflecting a major evidence gap in this domain. Second, economic evaluations are scarce; no trial to date has incorporated cost-effectiveness or cost-utility analyses, which are crucial for informing reimbursement policies and national screening strategies [25]. Third, evidence on long-term outcomes such as interval cancer rates, stage at detection, or mortality reduction remains absent, limiting our ability to assess AI’s impact beyond short-term detection metrics. Finally, patient-centered outcomes have been underexplored: whether AI-supported workflows reduce diagnostic delays, anxiety, or improve trust and adherence to screening programs remains unknown. Addressing these gaps through pragmatic, multi-modality, longitudinal, and patient-focused trials will be essential for guiding safe and equitable AI integration into routine clinical practice.

## Conclusions

This systematic review demonstrates that AI has reached a pivotal stage in oncologic imaging, with evidence from randomized trials and large-scale real-world studies showing non-inferior or superior diagnostic accuracy compared to radiologists, alongside tangible operational benefits such as reduced workload, faster diagnostic timelines, and improved predictive value of recalls. While breast cancer screening currently has the most mature evidence base, early data in lung imaging also signal promising applicability across modalities.

The overarching take-home message is that AI is not a replacement for radiologists but a powerful augmentative tool that can enhance efficiency, maintain safety, and potentially expand access to high-quality screening, particularly relevant in contexts of workforce shortages and rising cancer burden. However, robust cost-effectiveness data, long-term outcomes, and patient-centered evaluations remain missing, underscoring the need for continued rigorous investigation. By synthesizing the most up-to-date and methodologically strong evidence, our study provides critical insights to inform clinicians, policymakers, and health systems about the safe and effective integration of AI into cancer screening pathways.
